# The Five Diaphragms in Osteopathic Manipulative Medicine: Myofascial Relationships, Part 2

**DOI:** 10.7759/cureus.7795

**Published:** 2020-04-23

**Authors:** Bruno Bordoni

**Affiliations:** 1 Physical Medicine and Rehabilitation, Foundation Don Carlo Gnocchi, Milan, ITA

**Keywords:** diaphragm, osteopathic, fascia, myofascial, fascintegrity, physiotherapy

## Abstract

The article continues the anatomical review of the anterolateral myofascial connections of the five diaphragms in osteopathic manipulative medicine (OMM), with the most up-to-date scientific information. The postero-lateral myofascial relationships have been illustrated previously in the first part. The article emphasizes some key OMM concepts; the attention of the clinician must not stop at the symptom or local pain but, rather, verify where the cause that leads to the symptom arises, thanks to the myofascial systems. Furthermore, it is important to remember that the human body is a unity and we should observe the patient not as a series of disconnected segments but as multiple and different elements that work in unison; a dysfunction of tissue will adversely affect neighboring and distant tissues. The goal of the work is to lay solid foundations for the OMM and the five-diaphragm approach showing the myofascial continuity of the human body.

## Introduction and background

The approach to the five diaphragms in osteopathic manipulative medicine (OMM) is part of the respiratory-circulatory model, whose principle is the free movement of body fluids to maintain or improve patient health [1-2]. The OMM philosophy is based on patient-centred care, applying scientific knowledge and clinical experience [3-4]. The use of the five-diaphragm model is part of the OMM philosophy and represents one of the many manual therapeutic choices to guide the patient towards his maximum expression of well-being. Treating one or more body diaphragms is an osteopathic therapeutic option: tentorium cerebelli; tongue; thoracic outlet; diaphragm; pelvic floor. Other diaphragms are not considered, such as the sole of the foot or the posterior area of the knee or other body portions reminiscent of a diaphragm, because the five diaphragms are vital for survival; without the limbs, a person can live but, without the five diaphragms, a person risks death. Not surprisingly, working with this orientation can make a difference in the patient's clinical picture [5-9]. The article reviews the anatomy of the anterolateral myofascial relationships of the five diaphragms, continuing and concluding the path of the first part of the previous article.

## Review

Systemic myofascial relationships of the five diaphragms: anterolateral area

**Figure 1:**
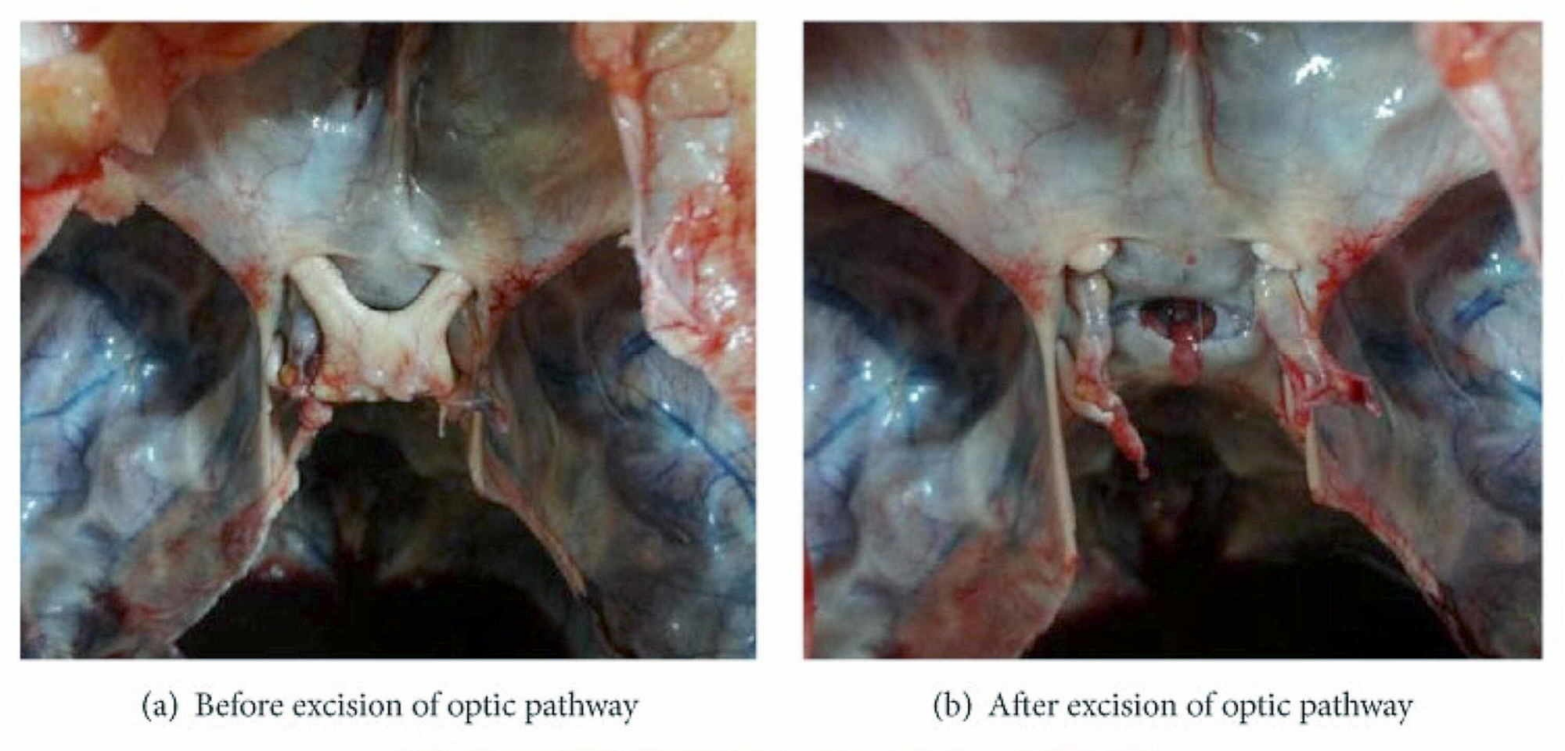
The images show the close relationship between the optic chiasma, the optic nerve, and the diaphragma sellae Figure A shows the presence of the optic chiasm covering diaphragma sellae; figure B shows the foramen of the diaphragma sellae. Images reproduced with permission of Dr. Doris George Yohannan [14].

There are several ligamentous and fascial structures not always studied and understood at a sub-occipital level, such as the sub-occipital ligament (between the occipital condyles) or Gerber's ligament (near the cruciform ligament) [10-11]. Probably, because they are not always present and found during anatomical dissections. Other internal ligaments at the base of the skull are poorly studied due to the difficulty of access but they are always present and connected to the meningeal structures, such as bridges between the posterior and anterior portion of the tentorium cerebelli: interclinoid; caroticoclinoid or anterior interclinoid; posterior petroclinoid; petrosphenoid or Grüber’s ligament; pterygospinous or Civinini ligament; pterygoalar or Hyrtl-Calori or innominate ligament [12]. Another element that connects the dural system is the optic nerve. Its coating is dural and bilaminar (in adults) [13]. The diaphragma sellae derives from the dural continuation of the tentorium cerebelli; the optic chiasm passes over the diaphragma sellae, sharing dural fibers (Figure 1) [14]. There is a close dural relationship between the tentorium cerebelli and the optic nerve.

**Figure 2:**
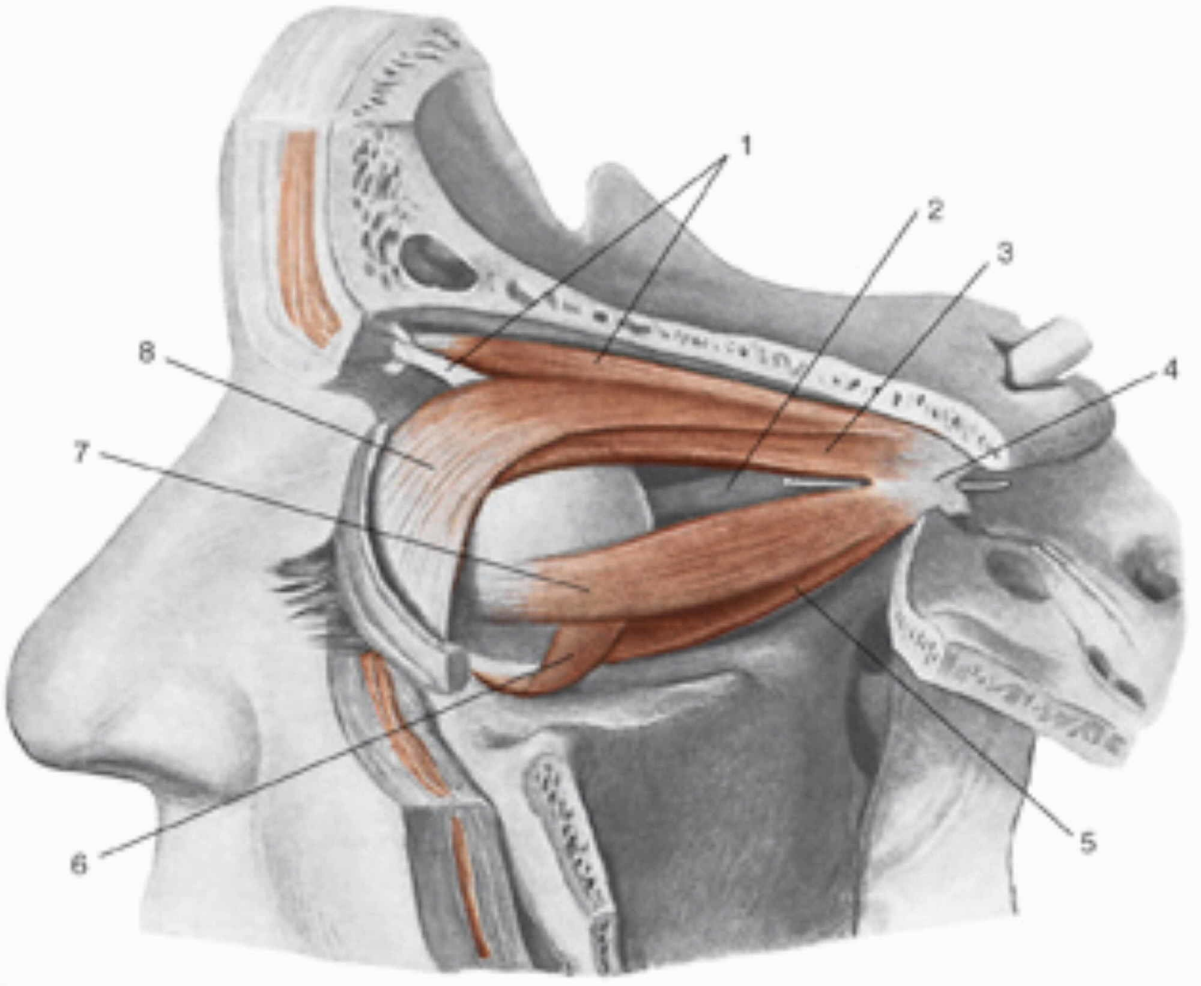
1, Superior oblique muscle; 2, optic nerve; 3, superior rectus muscle; 4, tendinous ring of Zinn; 5, inferior rectus muscle; 6, inferior oblique muscle; 7, lateral rectus muscle; 8, superioris levator palpebrae muscle. Reproduced with permission from Anastasi G, et al., Anatomia dell’uomo, fourth edition [Human Anatomy], vol 3, 2010, Milan: Edi-Ermes, p. 174.

**Figure 3:**
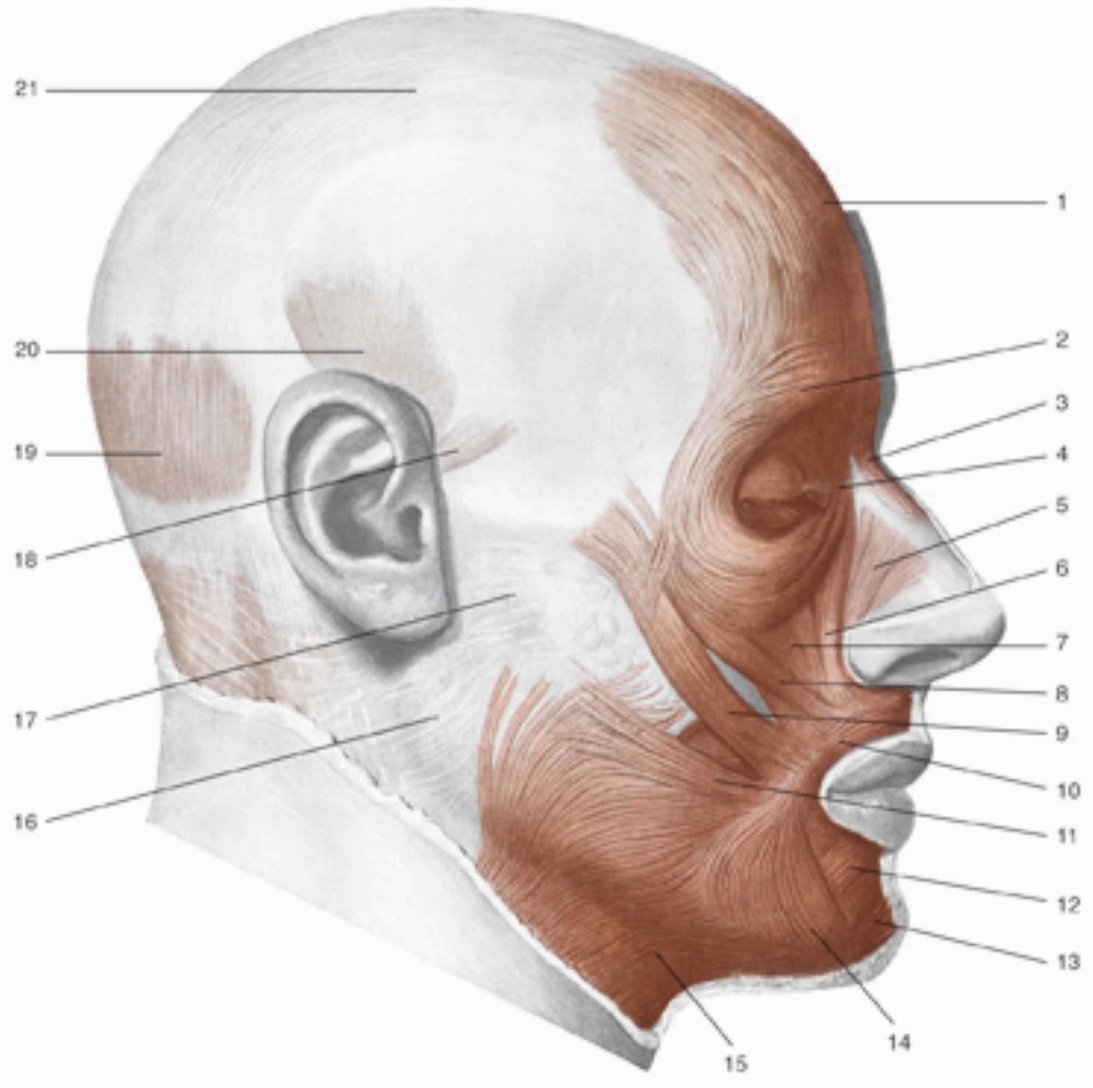
1, Frontal muscle; 2, orbicularis oculi muscle, part of the eyelid; 3, procerus muscle; 4, medial palpebral ligament; 5, nasal muscle; 6, levator muscle of the upper lip and of the wing of nose muscle; 7, levator of the upper lip muscle; 8, small zygomatic muscle; 9, great zygomatic muscle; 10, orbicularis oris muscle; 11, risorio muscle; 12, squared muscle of the inferior lip; 13, mentalis muscle; 14, triangular muscle; 15, platysma muscle; 16, parotid fascia; 17, fascia masseter; 18, anterior auricular muscle; 19, occipital muscle; 20, temporoparietal and upper auricular muscles; 21, aponeurotic galea. Reproduced with permission, from Anastasi G, et al., Anatomia dell’uomo, fourth edition [Human Anatomy], 2010, Milan: Edi-Ermes, p. 127.

In its path, the optic nerve merges with the Tenon capsule which, as previously described, is in continuum with the myofascial system that leads to the sub-occipital area and to the dura mater [2]. The extraocular muscles attach themselves to the Tenon capsule and for myofascial continuity they communicate with the neighboring muscles, involving the contractile districts of the face: procerus; orbicularis oculi; levator labii superioris alaeque nasi; zygomatic muscles (upper and lower); levator labii superioris; malaris; levator anguli oris; depressor labii inferioris; depressor anguli oris [15]. All the muscles of the face act in perfect coordination and fascial continuity. The anterolateral area of the face, the cheek, involves some of the muscles mentioned (orbicularis oculi with its lower portion, levator labii superioris alaeque nasi, levator labii superioris, zygomatic muscles, risorius, levator anguli oris); the masseter muscle stands out among the cheek muscles, partially covered by the buccinator muscle. There is a link of myofascial continuity of the buccinator muscle with the tendinous area of the temporalis muscle (Figures 2-3) [16].

**Figure 4:**
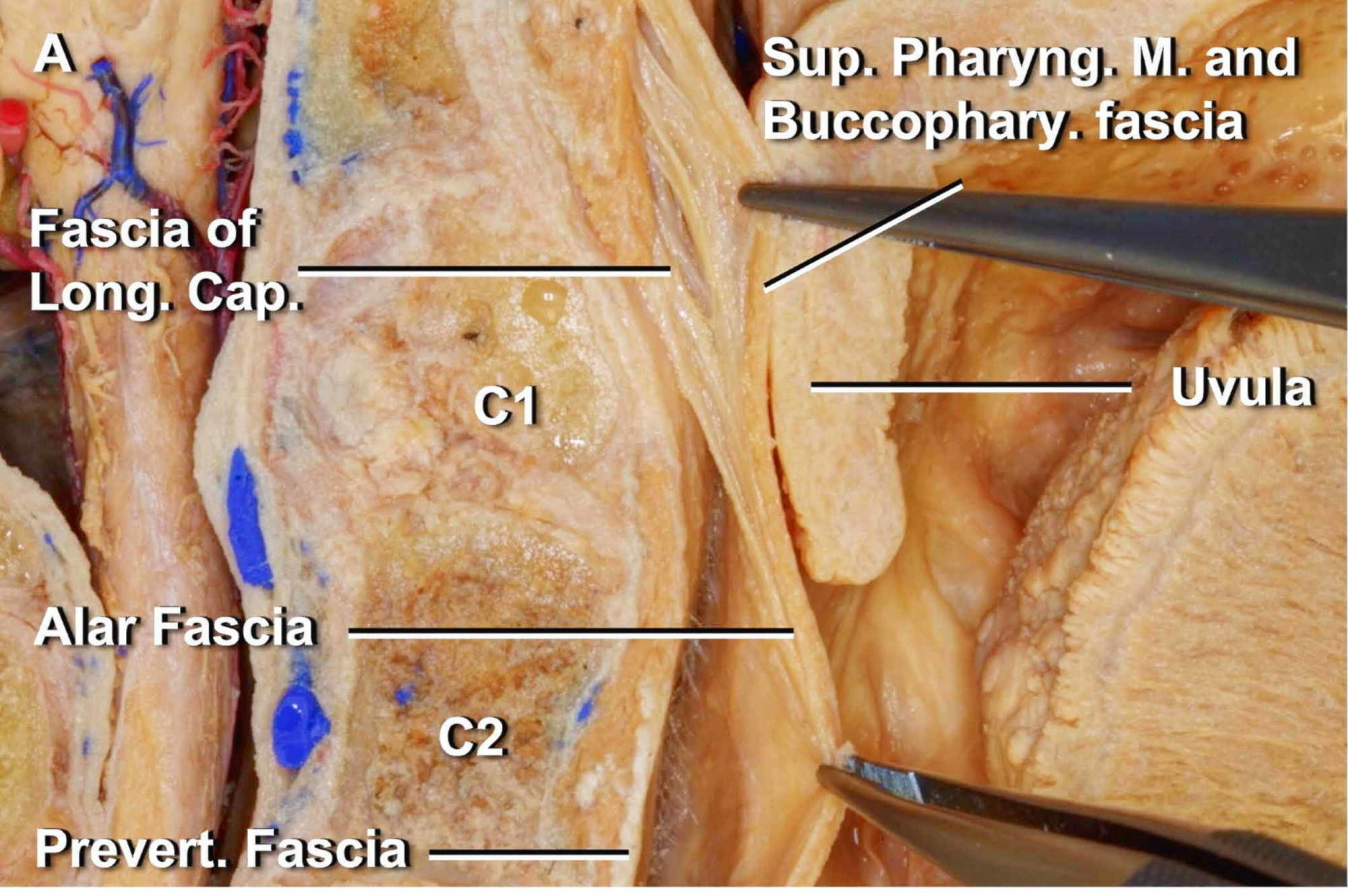
The alar fascia is identified behind the buccopharyngeal fascia below the C1 level and fuses to the fascia of the longus capitis lateralis muscle above the C1 level. The tongue is seen in its most proximal portion in the figure, on the far right. The image is reproduced with permission of Dott Noritaka Komune [21].

The buccinator muscle via the pterygomandibular raphe is in myofascial communication with the tensor veli palatini, with the superior pharyngeal constrictor, the masseter and the mylohyoid (or buccal floor) [17-18]. The interpterygoid fascia or temporopterygoid fascia arises from the base of the skull (sphenoid) and connects the pterygoid muscles, involving the insertion of the muscle temporalis, playing an important role for the delimitation of the pterygomandibular space, which ends above the hyoid bone [19-20]. The interpterygoid fascia merges with the main muscle of the tongue (styloglossus) and with the fascial system of the internal carotid artery [21]. The interpterygoid fascia is in direct contact with the sphenomandibular and stylomandibular ligaments [19]. The muscles of the face, including the chewing muscles, are interconnected with each other, with the base of the skull, with the hyoid bone and the lingual complex (Figure 4).

**Figure 5:**
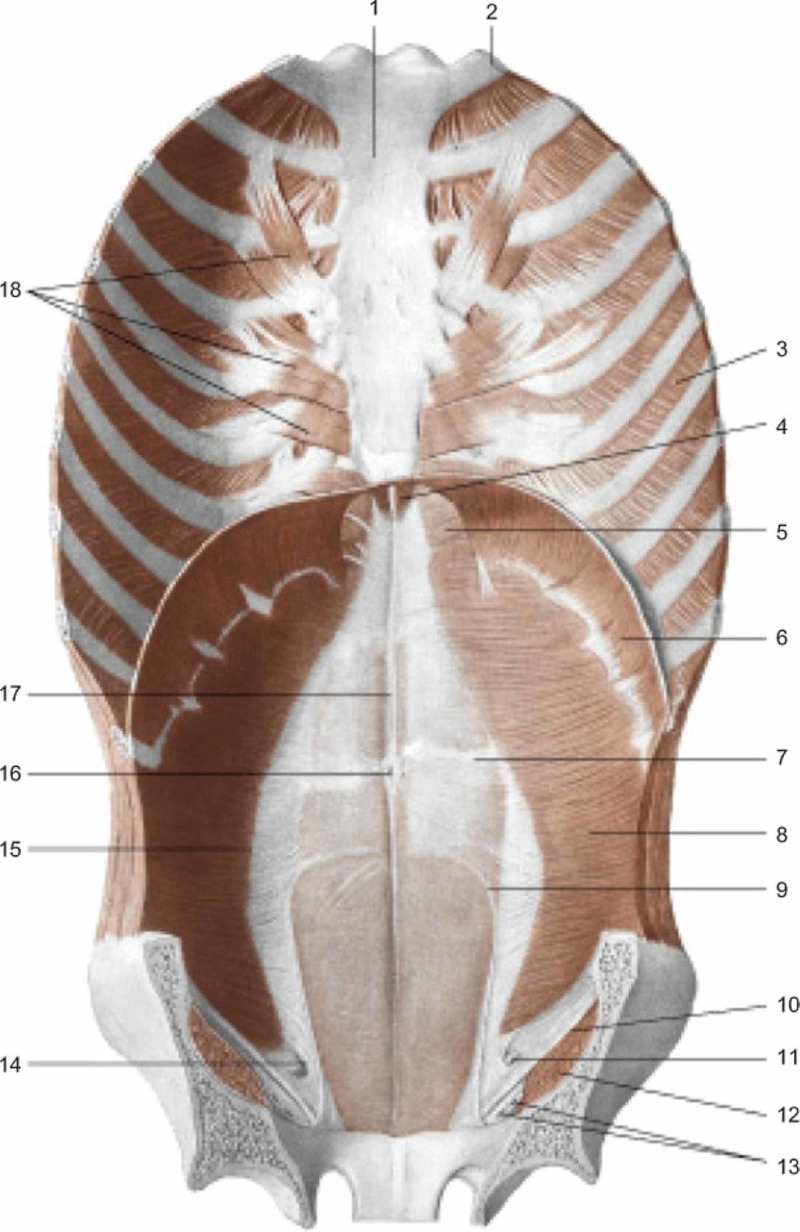
Muscles of the anterior wall of the trunk; view from inside 1: sternum; 2: first rib; 3: internal intercostal muscle; 4: sternal part of the diaphragm; 5: sternocostal trigon; 6: costal part of the diaphragm; 7: tendinous sign of the rectus abdominis muscle; 8: transversus abdominis muscle; 9: arcuate line; 10: inguinal ligament; 11: spermatic cord; 12: iliopsoas muscle; 13: the femoral vessels; 14: deep inguinal ring; 15: semilunar line; 16: umbilicus; 17: linea alba; 18: transverse muscle of the chest. Reproduced with permission, from Anastasi G, et al., Anatomia dell’uomo, fourth edition [Human Anatomy], 2010, Milan: Edi-Ermes, p. 174.

The superficial facial and cervical connective tissue forms the parotid fascia (with the masseteric fascia, temporal fascia), involving the styloid muscles (stylopharyngeus, stylohyoid, styloglossus), the platysma and digastric muscle, the sternocleidomastoid muscle (SCM) [22]. The anatomy demonstrates the myofascial continuity between the anterior and posterior parts of the body, involving the tentorium cerebelli, the lingual complex and the thoracic outlet. The superficial and deep cervical fascias surround the viscera and the suprahyoid and infrahyoid muscles, to flow anterolaterally to the superficial muscles of the neck (SCM, subclavian muscle, platysma), to the clavicle, the sternal body; at the thoracic outlet level the fasciae divide to cover the anterior (fascia thoracis superficialis) and deep chest area (as described in the previous first part [23]. The thoracis superficialis fascia covers the pectoralis major muscle, the pectoralis minor, the serratus, the sternalis (when present), and is connected to the lateroposterior musculature since the superficial fascia is the same [24-25]. The deep fascia of the neck before entering the mediastinum, at the cervical level, covers the thyroid posteriorly, while anteriorly and laterally the superficial fascia covers the thyroid, the thyroid muscles, the Berry ligament (between the thyroid and the laryngeal-tracheal complex); the superficial fascia builds the lingual frenulum (between the arch of the jaw and the buccal floor), involving the sublingual glands and submandibular ducts [26-27]. The sternothyroid muscle covers the surface of the lateral fascia of the thyroid while, anteriorly, the thyroid comes into contact with the proximal portion of the omohyoid muscles and the sternohyoid; the SCM stands between the latter two muscles in its anterior portion. Returning to the thoracic outlet, the fascial continuum covering the pectoral and thoracic muscles involves the deltoid and posterior musculature (infraspinatus fascia, deltoid fascia), as well as the fasciae of the upper limb [28-29]. The pectoralis fascia that covers the pectoralis major muscle continues, covering the sternum and passing to the contralateral muscle; distally at the level of the xiphoid process, the fascia pectoralis continues to merge with the abdominal fascia, on the same side and on the contralateral side [29]. There is no fascial discontinuity between the superficial and deep bands of the neck with the fascia pectoralis and the lateral and posterior muscles of the trunk (deltoid muscles, serratus anterior muscles, pectoralis minor muscles, teres major muscles, subscapularis muscles, trapezius muscle, latissimus dorsi muscles) [29]. The pectoralis major continues with the abdominal fascia, including the aponeurosis of the external oblique; the abdominal fascia covering the rectus abdominals involves the rib cartilages (V-VII) and the pubic symphysis. The abdominal fascia and the underlying abdominal muscles (rectus abdominis, obliques and transversus abdominis), together with the transversalis fascia, communicate with fascial continuity with the thoracolumbar fascia (Figure 5) [25].

The transversalis fascia (TF) is the continuation of the endothoracic fascia and stands between the epimysium of the diaphragm muscle and the underlying viscera. In its path, TF is found under the epimysium of the transversus abdominis and above the parietal peritoneum; behind the TF there is also the renal lodge, where it merges with the Gerota fascia (anterior) and with the Zuckerckandl fascia (posterior to the kidney) [30-31]. TF contacts the Zuckerckandl fascia and through the latter, comes into contact with the prevertebral fascia; laterally, TF comes into contact with the quadratus lomborum muscle [31]. TF forms the connective tissue where the rectus abdominis and the oblique muscles rest and envelop themselves, as well as the transversus abdominis. The outermost portion of the TF is the alba line where, at the level of the umbilical area, it merges with some fascial structures connected to the liver: round ligament or ligamentum teres hepatis; medial umbilical ligaments; median umbilical ligament or urachus [32]. The urachus at the inguinal ring merges below to strengthen the spermatic cord in the inguinal canal or to contribute to the formation of the common vaginal tunic [33]. Vestigial fascial folds or lateral umbilical folds can also be found [29]. TF encounters another structure called ligamentum venosum (between left portal vein and left hepatic vein) [34]. TF merges in part with the iliac fascia (deriving from the psoas and iliacus muscles) in its anteroinferior and anteromedially portion; near the inguinal ligament; TF and iliac fascia merge with pectineal fascia [33]. The pectineus muscle is in close contact with the pectineal ligament (ventrally and superiorly), which is connected to the inguinal ligament via the lacunar ligament; TF binds in the iliopubic portion to this complex [35]. At the iliopubic level, TF and its more superficial expansions bind to Henle's inguinal falx, which acts as a bridge to the inguinal ligament [36]. A fascial thickening of TF that passes medially through the deep inguinal ring is known as the Hesselbach ligament or semilunar fold of the transverse fascia or interfoveolar ligament [37]. The posterior face of the inguinal ligament (IL) binds frontally to TF by means of a fascial Thompson layer. There is a strong link between TF and IL. This relationship drags TF to involve the contralateral lower limb; the medial portion of the fascia lata derives from IL, whose fibers derive from TF [33, 38]. The connective tissue system of TF is contacted caudally with the adductor longus muscle; the pyramidalis muscles are below the TF attack (which covers the anterior pubic ligament) at the level of the pubic symphysis and merge with the adductor longus muscle [38]. The thoracolumbar fascia continues with the lower and upper limbs, as well as the anterior fascias from the skull to the pelvis and vice versa, making the human body a continuum without interruption; each body segment seen as a diaphragm (pelvis, respiratory diaphragm, thoracic outlet, tongue, and tentorium cerebelli) with their myofascial connections and with different vectors influence local and / or distant movements [39]. The subdivision into fascial layers is only an anatomical and didactic convention but, in reality, all the tissues are interconnected: " . . . unreasonable to apply the terms the superficial layer and deep layer, which are for the two-dimensional concept, to the three-dimensional clinical use [40]”.

Clinical reflections

The five diaphragms and the OMM remind us that the symptom must be seen in the whole body and not only as a localized anatomical structure. In fact, a chronic physical disorder also leads to dysfunctions in the psychological sphere [41]. There are many chronic pathologies where a disturbance of one of the diaphragms leads to multiple comorbidities, and hence the clinical motivation in manually treating the diaphragm(s) with an osteopathic approach [1-2,7-8, 41-43]. We also remember the nervous system, which not only communicates with the whole body but passes through the myofascial system. The vagus nerve is wrapped in the deep cervical fascia, the same fascia that involves the suboccipital muscles [23]. The vagus nerve innervates the subtentorial portion and the crural area of the diaphragm (esophageal hiatus) and with the presence of parasympathetic nerve endings to the pelvic tissues [1-2]. Considering that nervous tissue is a highway of electrical and biochemical information, capable of moving this information both towards the periphery and towards the medulla or the central nervous system, a dysfunction of the myofascial system can be one of the causes of chronic pain that involves more diaphragms: headache, alteration of the relationship between esophagus and diaphragm, impaired swallowing, brachialgia, pelvic pain [44-48]. The phrenic nerve and the sympathetic trunk near the vertebral bodies are enveloped by the prevertebral fascia [49]. The sympathetic system and phrenic nerve are able to carry somatic and visceral nociceptive afferent information; their structural and functional alteration, such as an abnormal compression of the cervical tract that affects the fascial system, or in other anatomical areas where they pass, can trigger multiple symptoms [1-2, 43, 50]. The five diaphragms are a strategy that can be used, as for other osteopathic medicine strategies, which is based on the fascial continuum and the neurological continuum. When we inhale the tongue it performs a retrusion action where the diaphragm is lowered as well as the pelvic floor; this happens thanks to the intervention of the supramedullary centres of the breath (nucleus retroambiguus), which govern the mentioned structures [1-2]. A correct level crossing of the thoracic outlet is fundamental for the nerve structures that will affect the remaining diaphragms, and a correct dural tension of the tentorium cerebelli will be important to avoid cascade disturbances as well [1-2].

## Conclusions

The second part of the article illustrated the myofascial connections of the anterolateral area of the body, showing continuity with the posterolateral myofascial system. OMM has several manual strategies to deal with the multiple disorders of patients, and the five diaphragm approach is one of these strategies. The article, divided into two parts, contains scientific information on myofascial body connections that have never been collected and mentioned in a single work. The hope is that the text will become an excellent reference point for clinicians who study and work with osteopathic medicine.
